# IL-17B/IL-17RB signaling cascade contributes to self-renewal and tumorigenesis of cancer stem cells by regulating Beclin-1 ubiquitination

**DOI:** 10.1038/s41388-021-01699-4

**Published:** 2021-03-01

**Authors:** Qingli Bie, Hui Song, Xinke Chen, Xiao Yang, Shuo Shi, Lihua Zhang, Rou Zhao, Li Wei, Baogui Zhang, Huabao Xiong, Bin Zhang

**Affiliations:** 1 Department of Laboratory Medicine, Affiliated Hospital of Jining Medical University, Jining Medical University, Jining, Shandong PR China; 2 Institute of Forensic Medicine and Laboratory Medicine, Jining Medical University, Jining, Shandong PR China; 3 Department of General Surgery, Affiliated Hospital of Jining Medical University, Jining, Shandong PR China; 4 Institute of Immunology and Molecular Medicine, Jining Medical University, Jining, Shandong PR China

**Keywords:** Cancer stem cells, Gastrointestinal cancer

## Abstract

Cancer stem cells (CSCs) are characterized by robust self-renewal and tumorigenesis and are responsible for metastasis, drug resistance, and angiogenesis. However, the molecular mechanisms for the regulation of CSC homeostasis are incompletely understood. This study demonstrated that the interleukin-17 (IL-17)B/IL-17RB signaling cascade promotes the self-renewal and tumorigenesis of CSCs by inducing Beclin-1 ubiquitination. We found that IL-17RB expression was significantly upregulated in spheroid cells and Lgr5-positive cells from the same tumor tissues of patients with gastric cancer (GC), which was closely correlated with the degree of cancer cell differentiation. Recombinant IL-17B (rIL-17B) promoted the sphere-formation ability of CSCs in vitro and enhanced tumor growth and metastasis in vivo. Interestingly, IL-17B induced autophagosome formation and cleavage-mediated transformation of LC3 in CSCs and 293T cells. Furthermore, inhibition of autophagy activation by ATG7 knockdown reversed rIL-17B-induced self-renewal of GC cells. In addition, we showed that IL-17B also promoted K63-mediated ubiquitination of Beclin-1 by mediating the binding of tumor necrosis factor receptor-associated factor 6 to Beclin-1. Silencing IL-17RB expression abrogated the effects of IL-17B on Beclin-1 ubiquitination and autophagy activation in GC cells. Finally, we showed that IL-17B level in the serum of GC patients was positively correlated with IL-17RB expression in GC tissues, and IL-17B could induce IL-17RB expression in GC cells. Overall, the results elucidate the novel functions of IL-17B for CSCs and suggest that the intervention of the IL-17B/IL-17RB signaling pathway may provide new therapeutic targets for the treatment of cancer.

## Introduction

Gastric cancer (GC) is the fourth most common malignancy and the second leading cause of cancer-related death worldwide [1]. GC has remained a deadly disease mainly because it is characterized by a poor overall survival rate, frequent relapse, metastasis, and chemotherapy resistance [2]. The cancer stem cell (CSC) hypothesis posits the existence of minor populations of CSCs that are uniquely capable of seeding new tumors, and CSCs have attracted considerable attention in recent years [3]. Accumulating evidence indicates that CSCs can self-renew and differentiate into multiple lineages contributing to tumor metastasis, aggressiveness, recurrence, and drug resistance [4]. Thus, considering the crucial role of CSCs, it is urgent to distinguish CSCs from the bulk population of non-CSCs, to explore novel therapeutic strategies for eradicating CSCs.

The interleukin-17 (IL-17) superfamily consists of six ligands (IL-17A through IL-17F) and their corresponding receptors (IL-17RA through IL-17RE). The *IL-17B* gene is located on human chromosome 5q32-34, and IL-17B functions by binding to its specific receptor IL-17RB to activate downstream signals [5]. Huang et al. were the first to report that IL-17RB is highly expressed in breast cancer tissues, and autocrine- or paracrine-derived IL-17B significantly promotes the tumorigenicity of breast cancer [6]. They subsequently confirmed that the metastatic ability of pancreatic cancer cells was significantly inhibited by blocking IL-17B/IL-17RB signaling with monoclonal antibodies that targeted IL-17RB [7]. However, it is unclear whether the biological functions of IL-17B are elicited through its direct effects on cancer cells or CSCs.

Our previous studies revealed that IL-17RB is highly expressed in GC tissues and is closely associated with the prognosis of GC [8]. The research has implied a crucial role of the IL-17B/IL-17RB signaling cascade in tumor biology. In liver cancer, IL-17E secreted by non-CSCs combined to IL-17RB on CSCs and promoted the self-renewal capacity of CSCs [9]. Transplanted Thy1-positive cells induced the self-renewal of small hepatocyte-like progenitor cells and inhibited their differentiation by mediating IL-17RB signaling [10]. These findings suggest that IL-17RB-mediated signaling could play a key role in stem-cell homeostasis. However, the biological functions of IL-17B and the activation of the IL-17B/IL-17RB signaling pathway in CSCs need to be further elucidated.

Autophagy is the regulatory mechanism of the cell through which unnecessary or dysfunctional components are eliminated. Accumulating evidence indicates that autophagy is involved in the homeostasis of CSCs and contributes to the regulation of CSCs in terms of self-renewal, distant metastasis, tumorigenesis, drug resistance, and angiogenesis [11, 12]. Li et al. found that disrupting Beclin-1 expression inhibited stem-cell-like properties and restored sensitivity to osimertinib cytotoxicity [13]. Autophagy also regulates the chemoresistance of GC-CSCs by activating Notch signaling [14]. Autophagy-related 4A cysteine peptidase (ATG4A), an autophagy-regulating molecule, induces the epithelial–mesenchymal transition (EMT) and certain stem-like properties in gastric cells [15]. These previous findings have revealed that the activation of autophagy is crucial in the malignant biological behaviors of CSCs. However, the signals causing autophagy activation in CSCs are poorly understood.

In the present study, we demonstrated that IL-17RB was highly expressed in GC-CSC-like cells. Recombinant IL-17B (rIL-17B) promoted the sphere-formation ability of CSCs in vitro and enhanced tumor growth and metastasis in vivo. Furthermore, the activation of autophagy was critically involved in IL-17B/IL-17RB-mediated regulation of CSC functions. Therefore, the results reveal novel functions of IL-17B for CSCs and implicate the importance of the IL-17B/IL-17RB signaling pathway in maintaining CSC homeostasis, suggesting that this pathway is a new therapeutic target for cancer.

## Results

### IL-17RB is highly expressed in CSCs and involved in tumor cell differentiation in GC tissues

**Fig. 1:**
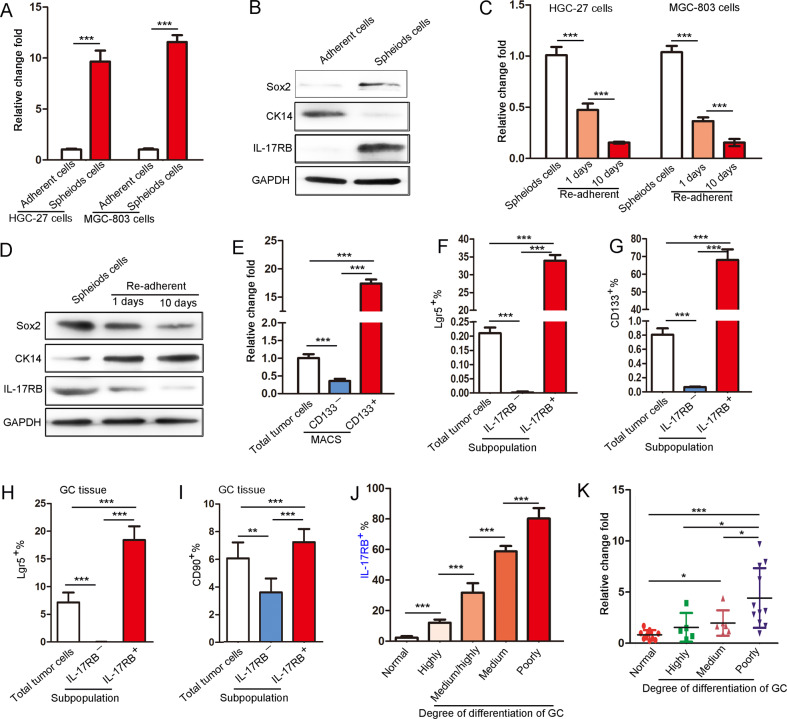
Expression of IL-17RB was upregulated in spheroid cells and was closely associated with the degree of GC tissue differentiation. **A** qRT-PCR was performed to detect the expression of *IL-17RB* in spheroid cells and normal adherent cultured HGC-27 and MGC-803 cells (Fig. S1A for acquisition; *n* = 3; ***p < 0.001). **B** The protein expression of CK14, Sox2, IL-17RB, and GAPDH in spheroid and normal adherent cultured cells was assayed using western blotting. **C** qRT-PCR was performed to detect *IL-17RB* in spheroid and re-adherent cultured HGC-27 and MGC-803 cells (Fig. S1D for acquisition; *n* = 3; ***p < 0.001). **D** The protein expression of CK14, Sox2, IL-17RB, and GAPDH in spheroid and re-adherent cultured cells was analyzed using western blotting. **E** qRT-PCR was used to detect *IL-17RB* mRNA in CD133^+^ and CD133^−^ HGC-27 cells isolated through magnetic bead sorting (*n* = 3; ***p < 0.001). **F**, **G** Statistical analysis of the proportion of Lgr5^+^ (**F**) and CD133^+^ (**G**) in IL-17RB^+^ and IL-17RB^−^ subsets of HGC-27 cells, examined through flow cytometry (*n* = 3; ***p < 0.001). **H**, **I** Statistical analysis of the proportion of Lgr5^+^ (**H**) and CD90^+^ (**I**) in IL-17RB^+^ and IL-17RB^−^ cell subsets in GC tissue, determined through flow cytometry (*n* = 3; ***p < 0.001). **J** Statistical analysis of the *p*roportion of IL-17RB positivity according to Fig. S3A (*n* = 6; ***p < 0.001). **K** qRT-PCR was conducted for analysis of *IL-17RB* mRNA expression in GC tissues with various degrees of differentiation (*n* = 22; *p < 0.05; ***p < 0.001).

Our previous study revealed that the IL-17B/IL-17RB signal promotes the growth and migration of tumor cells, and the expression of IL-17RB is positively correlated with the expression CSC markers [8]. However, the molecular mechanisms underlying the effects of IL-17B/IL-17RB signaling on CSC biological phenotypes are still not understood. To address this question, we generated spheroid cells from MGC-803 or HGC-27 cells by using serum-free medium (Supplementary Fig. S1A). We verified the spheroid cells by detecting CSC-associated markers’ high expression—including *OCT4*, *NANOG*, *SOX2*, and *LGR5*—using qRT-PCR. The expression of epithelial differentiation markers—including *MUC1* and *CK18*—was downregulated (Supplementary Fig. S1B, C). The expression of IL-17RB in the spheroid cells was 10-fold higher than that in adherent naturally growing cells, consistent with CSC markers’ expression pattern (Fig. 1A), and the results were verified through Western blot experiments (Fig. 1B). Based on these results, we speculated that IL-17RB expression in differentiated mature cells might be altered. Thus, we created a spheroid cell redifferentiation model by culturing spheroid cells in media containing 10% fetal bovine serum for 1 or 10 days [16] (Supplementary Fig. S1D). With the prolongation of the differentiation time (1–10 days), the expression of CSC markers significantly decreased, whereas differentiation markers’ expression increased (Supplementary Fig. S1E, F). As expected, the mRNA and protein expression of IL-17RB also decreased, consistent with the CSC markers’ expression pattern (Fig. 1C, D). To rule out the effect of culture medium and 2D vs. 3D culturing on IL-17RB expression, we purified CD133^+^ and CD133^−^ cells from HGC-27 cells through magnetic bead sorting, and the results revealed that IL-17RB mRNA expression in CD133^+^ HGC-27 cells was significantly higher than that in CD133^−^ cells (Fig. 1E). Next, we analyzed the expression of IL-17RB, Lgr5, and CD133 in GC cells by using flow cytometry (Supplementary Fig. S2A). Lgr5 and IL-17RB were co-expressed in the same cell population (Fig. 1F and Supplementary Fig. S2A), as were CD133 and IL-17RB (Fig. 1G and Supplementary Fig. S2A). These results suggest that IL-17RB represents a novel marker of GC-CSCs, prompting us to validate the results further using clinical specimens and in vivo experiments.

We detected the expression of IL-17RB, Lgr5, and CD90 in single tumor cells purified from GC tissues through flow cytometry (Supplementary Fig. S2B). As expected, only IL-17RB^+^ cells co-expressed Lgr5, while IL-17RB^−^ cells did not express Lgr5 (Fig. 1H and Supplementary Fig. S2B). Similarly, the percentage of CD90^+^ cells among IL-17RB^+^ cells was considerably higher than that among IL-17RB^−^ cells (Fig. 1I and Supplementary Fig. S2B). EMT is a key process in the malignant transformation in GC [17]. Mani et al. demonstrated that EMT-generated cells exhibit the properties of CSCs [18]. Thus, we hypothesized that the number of CSCs in tumor tissues might vary with the degree of differentiation of GC tissues. As predicted, the results showed that the expression of Sox2 was upregulated with the decrease of tumor differentiation degree, whereas CK18 expression displayed the opposite trend, as revealed by immunohistochemistry (Supplementary Fig. S3A). Notably, IL-17RB expression paralleled Sox2 expression, with the positive rates being ~18% in highly differentiated tissues, 38% in moderately highly differentiated tissues, 60% in moderately differentiated tissues, and 80% in poorly differentiated tissues (Fig. 1J and Supplementary Fig. S3A). A similar pattern of IL-17RB and Sox2 mRNA expression was also found in different differentiation degree of GC tissues as in immunohistochemistry (Fig. 1K and Supplementary Fig. S3B). In addition, positive correlations were observed between IL-17RB and Sox2 mRNA expression in GC tissues (Supplementary Fig. S3C). The analysis of clinical samples further confirmed that the IL-17RB-mediated signaling pathway might be critically involved in the regulation of CSC phenotypes.

### Activation of IL-17B/IL-17RB signaling augments the expansion of CSCs and sphere-formation ability

**Fig. 2:**
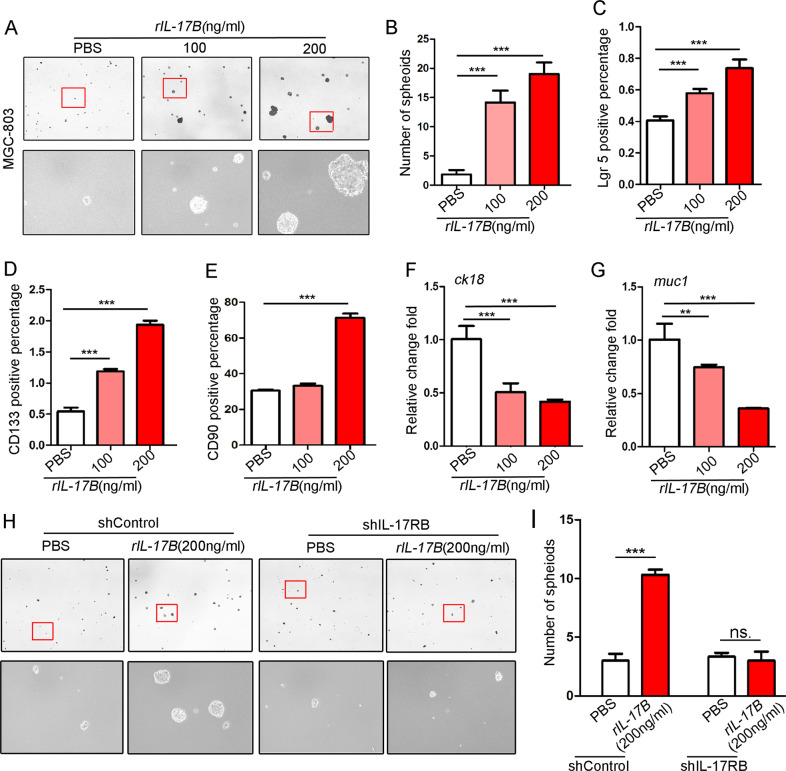
IL-17B promoted self-renewal and expansion of CSCs depending on IL-17RB. **A** Representative images of MGC-803 cells cultured in serum-free medium for 7 days and stimulated by rIL-17B or PBS. **B** Statistical analysis of the spheroid number (diameter > 50 μm) according to figure subpart A (*n* = 6; ***p < 0.001). **C–E** Flow cytometric analysis of the expression of Lgr5, CD133, and CD90 in MGC-803 cells treated with different concentrations of rIL-17B (*n* = 3; ***p < 0.001). **F**, **G** qRT-PCR was performed to detect the expression of *CK18* and *MUC1* in MGC-803 cells treated with different concentrations of rIL-17B (*n* = 3; **p < 0.01; ***p < 0.001). **H** Representative images showing the general number and size of spheroids in MGC-803 cells infected with shIL-17RB or shControl lentiviral vectors and stimulated with different concentrations of rIL-17B for 7 days. **I** Statistical analysis of the number of spheroids (diameter > 50 μm) based on figure subpart **H** (*n* = 6; ***p < 0.001).

To investigate how IL-17RB regulates the function of CSCs, we knocked down the expression of IL-17RB in MGC-803 or HGC-27 cells by using lentivirus-based shRNA (Supplementary Fig. S4A, B). Unexpectedly, interfering IL-17RB did not affect the sphere-formation ability of GC cells (Supplementary Fig. S4C), which might due to the low expression of IL-17B, a ligand of IL-17RB, in GC cells [5, 8]. Thus, we treated the cells with recombinant IL-17B (rIL-17B) to directly stimulate IL-17RB signaling in tumor cells. RIL-17B significantly promoted the sphere-formation ability of MGC-803 cells in a concentration-dependent manner (Fig. 2A, B); these results were also verified in HGC-27 cells (Supplementary Fig. S5A, B). Besides, we also found rIL-17B strengthened the cell cycle transformation from quiesecent phase to mitotic phase in GC cells (Supplementary Fig. S5C). Next, we discovered that rIL-17B significantly improved the proportion of cells positive for Lgr5, CD133, or CD90, and did so in a concentration-dependent manner except for the marker CD90 (Fig. 2C–E and Supplementary Fig. S6A–C). Conversely, rIL-17B inhibited the expression of *MUC1* and *CK18* mRNA (Fig. 2F, G). Significantly, knockdown of IL-17RB in MGC-803 cells reversed the sphere-formation ability induced by rIL-17B (Fig. 2H, I). The results suggest that IL-17B promotes GC’s cell stemness, dependent on IL-17RB expression.

### IL-17B promotes tumorigenesis and invasion of CSCs in vivo

**Fig. 3:**
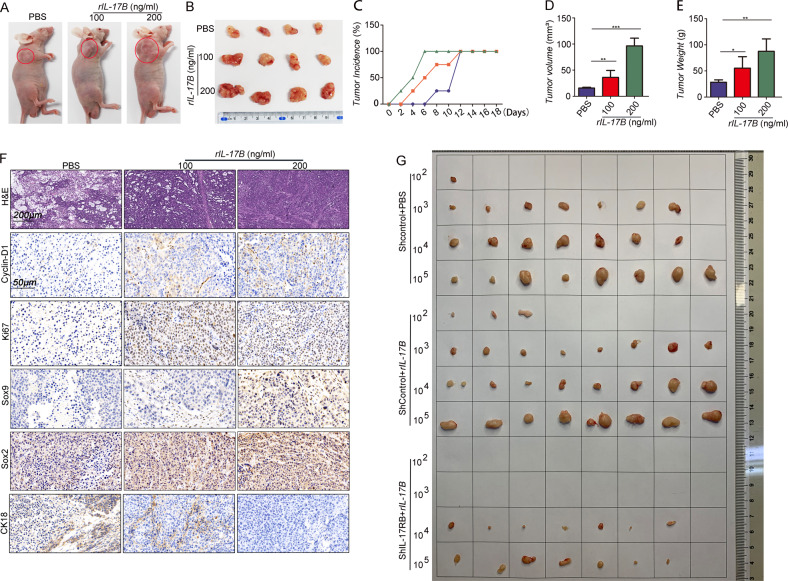
rIL-17B promoted the tumorigenic ability and growth of subcutaneous tumors of GC cells. **A** Representative images of subcutaneous tumors in nude mice. **B** Images of subcutaneous xenografts in nude mice. **C–E** The incidence rate (**C**), tumor volume (**D**), and tumor weight (**E**) were monitored for the indicated tumor (*n* = 4; *p < 0.05; **p < 0.01; and ***p < 0.001). **F** HE and immunohistochemical staining showed the expression of ki67, cyclin-D1, Sox9, Sox2, and CK18 in subcutaneous tumor tissues. **G** HGC-27-shIL-17RB cells treated with 200 ng/ml rIL-17B and HGC-27-shControl cells treated with 200 ng/ml rIL-17Bor PBS for 48 h, these cells were diluted and subcutaneously injected into nude mice. Tumors were examined over a 8 days period (*n* = 8 for each group).

CSCs possess the characteristics of fast tumor growth and high metastasis ability [19]. In the present study, to investigate the effect of IL-17B/IL-17RB signaling on GC cells in vivo, we established a xenograft tumor model through subcutaneous injection of MGC-803 cells treated with rIL-17B or not into nude mice. The results showed that rIL-17B promoted tumor growth in a concentration-dependent manner (Fig. 3A, B); with an increasing concentration of IL-17B, the tumorigenicity of the cells occurred earlier, and all tumors developed earlier in the mice (Fig. 3C). In addition, the tumor volume and weight increased in a concentration-dependent manner (Fig. 3D, E).

Morphological analysis showed that the cell distributions in tumor tissues from the mice injected with cells treated with rIL-17B were narrower than control cells (Fig. 3F). Moreover, the expression of proliferation markers, including ki67 and cyclin-D1, was significantly increased in groups treated with rIL-17B, and the expression of Sox9 and Sox2 was also significantly upregulated, as revealed by immunohistochemical analysis (Fig. 3F and Supplementary Fig. S7). However, the CK18 protein expression pattern displayed the opposite trend to the Sox9 and Sox2 expression (Fig. 3F and Supplementary Fig. S7). In addition, we performed a limiting dilution analysis experiment to further verify whether rIL-17B promote the stemness of GC cells. As shown in Fig. 3G, rIL-17B treatment promoted the tumorigenic frequency and tumor volume in HGC-27-shControl than PBS treatment in the same dilution concentration of cells, while the knockout of IL-17RB in HGC-27 cells obviously reversed the effection of rIL-17B pro-tumorigenic. And these two group (10^2^ and 10^3^ concentration HGC-27-shIL-17RB treated with rIL-17B) did not form tumors, tumor volumes of HGC-27-shIL-17RB treated with rIL-17B also were obviously less than HGC-27-shControl treated with rIL-17B in the same dilution cells. These results revealed that IL-17B enhances the growth and stemness of GC cells in vivo and the effect of IL-17B was mediated through IL-17RB.

**Fig. 4:**
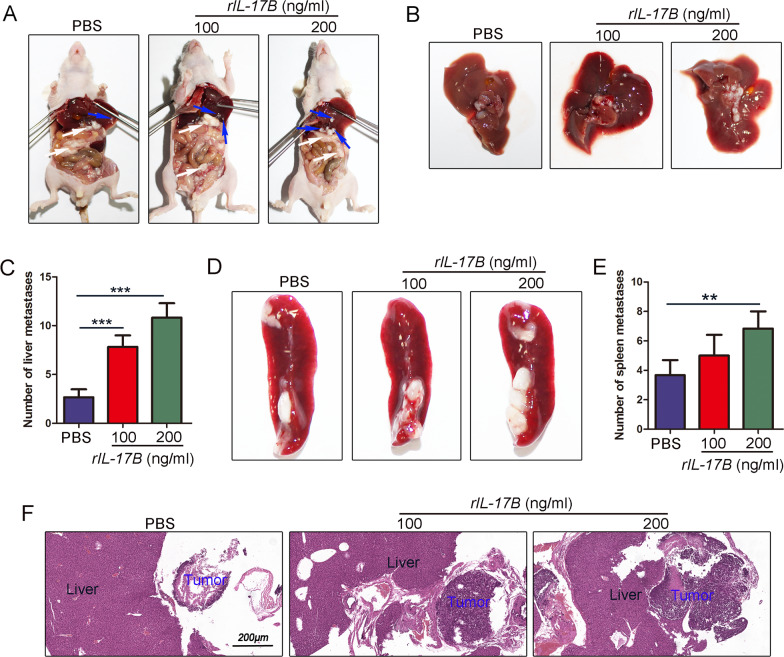
rIL-17B promoted peritoneal tumorigenesis and invasion ability of MGC-803 cells. **A** Representative images showing tumors in the peritoneal cavity of the mice injected with MGC-803 cells treated or not with rIL-17B for 48 h. **B** Macroscopic appearance of liver invasion. **C** The numbers of visible liver tumor invasion were statistically analyzed (*n* = 6; ***p < 0.001). **D** Macroscopic appearance of spleen invasion. **E** The numbers of visible spleen tumor invasion were statistically analyzed (*n* = 6; **p < 0.01). **F** HE staining showing liver tissues and invasion tumors.

Next, to verify the results as mentioned earlier, we established peritoneal tumorigenesis and experimental invasion model. In this model, we demonstrated that more tumors were formed in the abdominal cavity when the concentration of rIL-17B was higher (Fig. 4A). RIL-17B clearly induced liver invasion in a concentration-dependent manner (Fig. 4B, C). In addition, similar results were obtained for spleen invasion (Fig. 4D, E). Furthermore, histopathological staining showed that tumor nodules treated with rIL-17B invaded further into the inner layer of liver tissue, whereas these invasion were not found for phosphate-buffered saline (PBS)-treated tumor nodules, which remained near the liver (Fig. 4F). Thus, the in vivo results thus further corroborate that IL-17B/IL-17RB signaling strengthens the peritoneal tumorigenesis and invasive abilities of tumor cells.

### IL-17B stimulates autophagy activation in CSCs

**Fig. 5:**
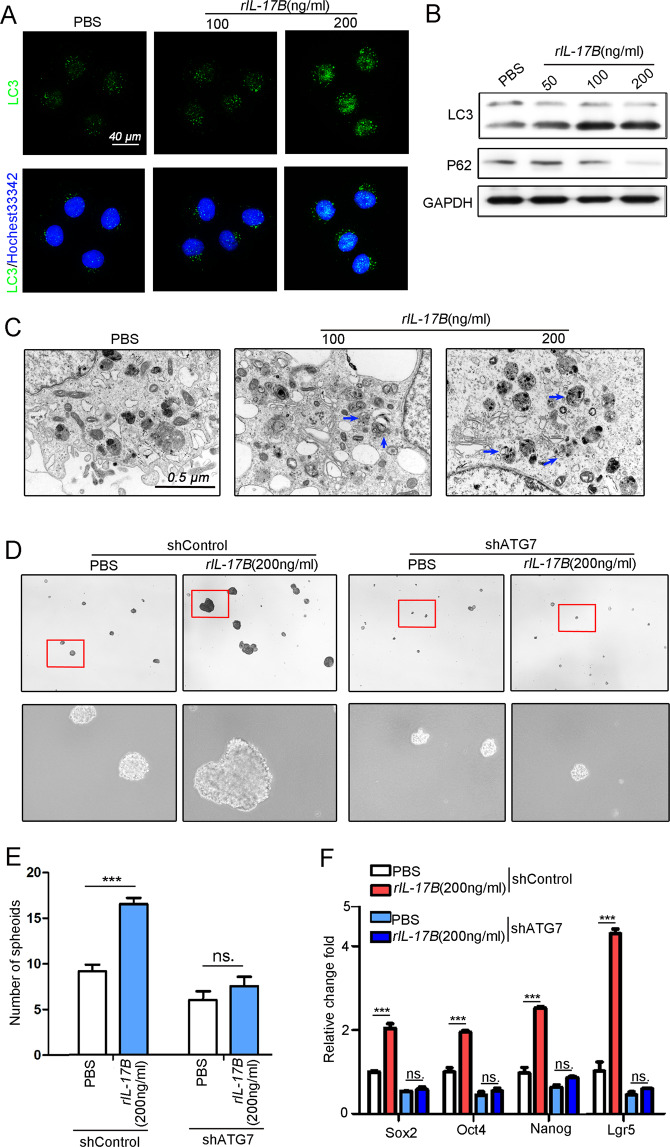
rIL-17B promoted self-renewal of GC cells by activating autophagy. **A** Confocal microscopy was used to detect LC3 protein expression in MGC-803 cells treated with rIL-17B or PBS. **B** Western blotting was used to assay the expression of LC3, p62, and GAPDH in MGC-803 cells treated with rIL-17B or PBS for 48 h. **C** TEM was used to analyze autophagosome formation in MGC-803 cells treated with rIL-17B or PBS for 48 h (blue arrow for autophagolysosome structure). **D** The spheroids in shControl or shATG7 lentiviral-infected MGC-803 cells treated with rIL-17B or PBS for 48 h were detected. **E** Spheroid numbers with diameter > 50 μm under ×40 microscope magnification were statistically analyzed (*n* = 6; ***p < 0.001; ns: not statistically significant). **F** qRT-PCR was used to detect *OCT4*, *NANOG*, *SOX2*, and *LGR5* in shControl or shATG7 lentiviral-infected MGC-803 cells treated with rIL-17B or PBS for 48 h (*n* = 6; ***p < 0.001; ns not statistically significant).

To explore the molecular mechanisms involved in the effects of IL-17B on CSC functions, we performed transcriptome sequencing analysis of HGC-27 cells treated with rIL-17B (Series record GSE164038, for information on GEO linking and citing, please refer to: https://www.ncbi.nlm.nih.gov/geo/info/linking.html) (Supplementary Fig. S8A–C). The data showed that PI3K-AKT signal were the most difference in HGC-27 cells treated with rIL-17B (Supplementary Fig. S8B). Previous studies have revealed that stimulating the AKT signaling pathway strongly activates autophagy [20, 21]. Considering that autophagy activation is critical for CSC functions [22–24], we hypothesized that the IL-17B/IL-17RB signaling pathway might regulate CSC functions by directly targeting autophagy activation. Confocal images of LC3 showed that IL-17B induced autophagosomes’ formation in a concentration-dependent manner (Fig. 5A). Western blotting also indicated that IL-17B promoted the cleavage-mediated transformation of LC3 protein and enhanced the expression of LC3 II (14 kDa) (Fig. 5B). These results were also verified in the other cell types, including HGC-27 and 293T cells (Supplementary Fig. S9A, B). In addition, rIL-17B decreased the expression of the P62 protein in these cells (Fig. 5B and Supplementary Fig. S9A, B). Transmission electron microscopy (TEM) experiments also showed increased autophagosome formation in IL-17B-treated MGC-803 cells compared with control cells (Fig. 5C).

To validate the role of autophagy in CSC functions, we blocked autophagy activation through the knockdown of ATG7 expression [24]. The results showed that the knockdown of ATG7 expression decreased the cleavage-mediated transformation of LC3 and inhibited the stem-cell markers’ expression of Oct4 and Sox2 (Supplementary Fig. S9C). The results confirmed that autophagy plays a critical role in regulating CSC phenotypes. Furthermore, knockdown of ATG7 expression abrogated the self-renewal of CSCs induced by IL-17B (Fig. 5D, E). In addition, IL-17B enhanced the expression of several CSC markers—including *OCT4*, *SOX2*, *LGR5* and *NANOG*—in MGC-803 cells infected with shControl; however, IL-17B had no effects on expression of these genes in cells with ATG7 knockdown (Fig. 5F). Western blot experiments confirmed these results (Supplementary Fig. S9D). Alternatively, chloroquine, an autophagy inhibitor, also inhibited the transformation of LC3 and the self-renewal of CSCs induced by rIL-17B (Supplementary Fig. S10A–C). Altogether, the results suggest that IL-17B causes the activation of autophagy, which regulates CSC functions.

### IL-17B induces K63-mediated ubiquitination of Beclin-1 by mediating Beclin-1–TRAF6 interaction

The results, as mentioned above, prompted us to further examine the molecular mechanism through which IL-17B causes activation of autophagy, which promotes the self-renewal of CSCs. Transcriptome sequencing analysis of HGC-27 cells treated with IL-17B did not identify any molecules directly related to autophagy among the genes with significant alterations (Supplementary Fig. S8C). Therefore, we thought that post-transcriptional modifications of some proteins might be involved in autophagy activation. Shi and Kehrl reported that tumor necrosis factor receptor (TNFR)-associated factor 6 (TRAF6)-induced Lys63 (K63)-mediated ubiquitination of Beclin-1 is critical for autophagy activation in macrophages, which was induced by toll-like receptor 4 (TLR4) [25]. IL-17RB has some functions similar to TLR4 functions, such as causing inflammation through TRAF6 [5, 26]. Thus, we hypothesized that the activation of autophagy in GC cells by IL-17B/IL-17RB signaling might be mediated by Beclin-1 ubiquitination.

**Fig. 6:**
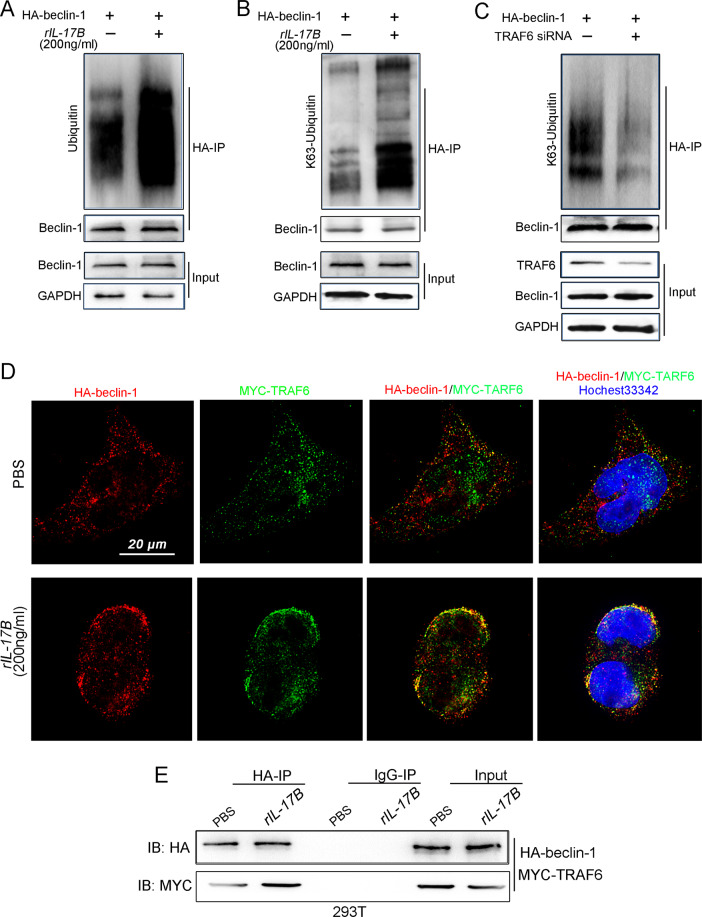
rIL-17B reinforced K63-mediated ubiquitination of Beclin-1 by promoting TRAF6 binding to Beclin-1. **A** IP showing the expression of ubiquitin, Beclin-1, or GAPDH in rIL-17B- or PBS-treated MGC-803 cells transfected with the HA-Beclin-1 plasmid and treated with MG132 (20 μM) for 6 h. **B** IP revealed lysine 63-linked ubiquitin, Beclin-1, or GAPDH in rIL-17B- or PBS-treated MGC-803 cells transfected with the HA-Beclin-1 plasmid and treated with MG132 (20 μM) for 6 h. **C** MGC-803 cells were transfected with HA-Beclin-1 and TRAF6 interference oligonucleotide sequence (TRAF6 siRNA). The indicated HA pull-down samples were analyzed using Western blotting for the presence of K63-linked ubiquitin. The expression of TRAF6, Beclin-1, and GAPDH was detected in the total protein (input). **D** Super-resolution microscopy was used to analyze the binding between TRAF6 and Beclin-1 in MGC-803 cells transfected with HA-Beclin-1 and the MYC-TRAF6 plasmid treated with rIL-17B or PBS for 48 h. **E** IP showing HA or MYC in rIL-17B- or PBS-treated MGC-803 cells transfected with HA-Beclin-1 and the MYC-TRAF6 plasmid.

As expected, the results revealed that IL-17B stimulation did induce Beclin-1 ubiquitination in MGC803 cells (Fig. 6A), and the protein and mRNA expression of Beclin-1 was unaffected (Supplementary Fig. S11A, B). Therefore, we assumed that the ubiquitination of Beclin-1 induced by rIL-17B was not mediated through the proteasome pathway. Moreover, we identified that IL-17B promoted the K63-mediated ubiquitination of Beclin-1 (Fig. 6B). One previous study revealed that TRAF6 is the major E3 ubiquitinligase that causes the K63-mediated ubiquitination of Beclin-1 [27, 28], were confirmed by the results in the present study. And we found that knockdown of TRAF6 expression reduced K63-mediated ubiquitination of Beclin-1 (Fig. 6C). Direct interaction between the E3 ubiquitinligase and the target protein is required for target protein ubiquitination. Thus, in the present study, we investigated whether the physical interaction between TRAF6 and Beclin-1 is regulated by IL-17B/IL-17RB signaling. Super-resolution confocal microscopy showed that IL-17B promoted the binding of TRAF6 to Beclin-1, and these three proteins were co-localized on the cell membrane (Fig. 6D). Coimmunoprecipitation (co-IP) experiments further confirmed that IL-17B helped TRAF6 bind to Beclin-1 (Fig. 6E). These findings suggest that IL-17B induces autophagy activation in CSCs through the K63-mediated ubiquitination of Beclin-1, which depends on the direct interaction between TRAF6 and Beclin-1. However, the precise mechanisms through which IL-17B initiates the direct binding of TRAF6 to Beclin-1 remain to be explored.

### IL-17RB is indispensable for autophagy activation

**Fig. 7:**
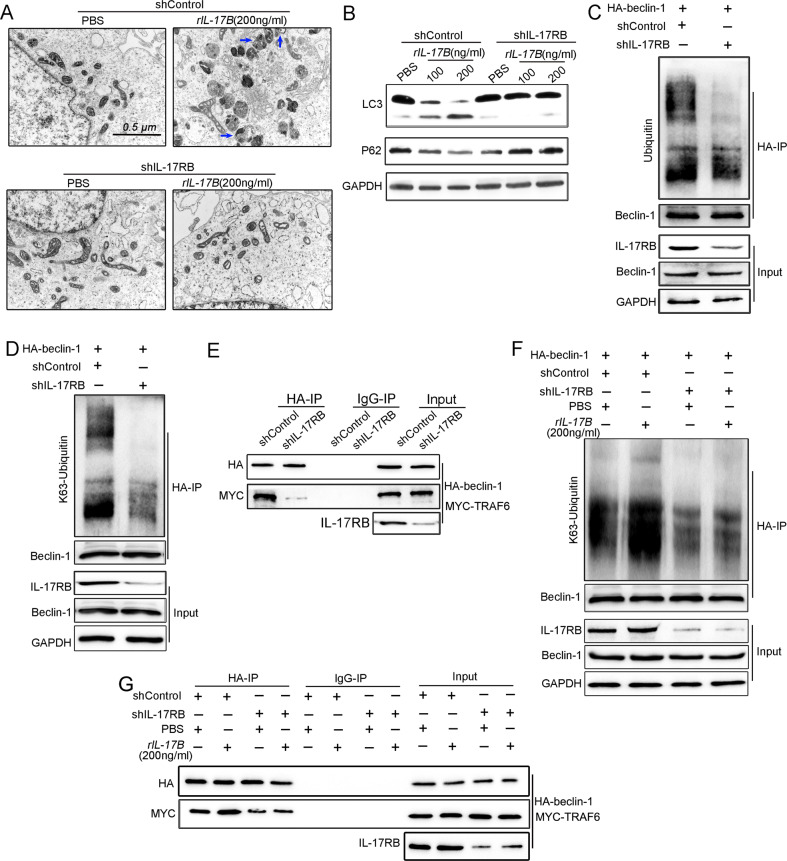
IL-17RB played a key role in activating autophagy and K63-mediated ubiquitination of Beclin-1 induced by rIL-17B. **A** TEM was used to detect autophagosome formation in rIL-17B- or PBS-treated MGC-803 cells infected with shControl or shIL-17RB lentiviral constructs. **B** The expression of LC3, P62, and GAPDH was assayed using western blotting in rIL-17B- or PBS-treated MGC-803 cells infected with shControl or shIL-17RB lentiviral constructs. **C**, **D** MGC-803 cells were transfected with the HA-Beclin-1 plasmid and subjected to IP by using an HA antibody or control IgG, followed by IB with ubiquitin/K63-linked ubiquitin, Beclin-1, IL-17RB, and GAPDH antibodies. **E** IP was used to analyze shIL-17RB or shControl MGC-803 cells that had been transfected with HA-Beclin-1 and the MYC-TRAF6 plasmid by using an HA antibody or control IgG, followed by IB with HA and MYC antibodies. **F** IP was used to analyze shIL-17RB or shControl MGC-803 cells treated with rIL-17B or PBS transfected with the HA-Beclin-1 plasmid by using an HA antibody or control IgG, followed by IB with K63-linked ubiquitin, Beclin-1, IL-17RB, and GAPDH antibodies. **G** IP was used to analyze shIL-17RB or shControl MGC-803 cells transfected with HA-Beclin-1 and the MYC-TRAF6 plasmid by using an HA antibody or control IgG with/without rIL-17B treatment, followed by IB with HA, MYC, and GAPDH antibodies.

The Fig. 6D shows the overlapping region of TRAF6 and Beclin-1 on the cell membrane after IL-17B treatment. IL-17RB is mainly expressed on the cell membrane [5]. Thus, we assumed IL-17RB is essential to autophagy activation and K63-mediated ubiquitination of Beclin-1 induced by IL-17B. In this study, TEM images showed that the knockdown of IL-17RB expression abrogated autophagy activation induced by IL-17B (Fig. 7A), reduced the conversion of LC3, and reversed the expression of P62 decreased by IL-17B (Fig. 7B); these results suggest the indispensable role of IL-17RB in causing autophagy activation. As expected, knockdown of IL-17RB expression decreased the K63-mediated ubiquitination of Beclin-1 (Fig. 7C, D). In addition, direct binding between TRAF6 and Beclin-1 was significantly reduced when IL-17RB expression was inhibited (Fig. 7E). In parallel, knockdown of IL-17RB expression abolished the K63-mediated ubiquitination of Beclin-1 induced by IL-17B (Fig. 7F). Furthermore, IL-17B mediated direct binding between TRAF6 and Beclin-1, regulated by IL-17RB (Fig. 7G). The results strongly suggest that IL-17RB is a crucial component in autophagy activation induced by IL-17B.

### IL-17B promotes IL-17RB expression, resulting in enhanced TRAF6–Beclin-1 interaction

**Fig. 8:**
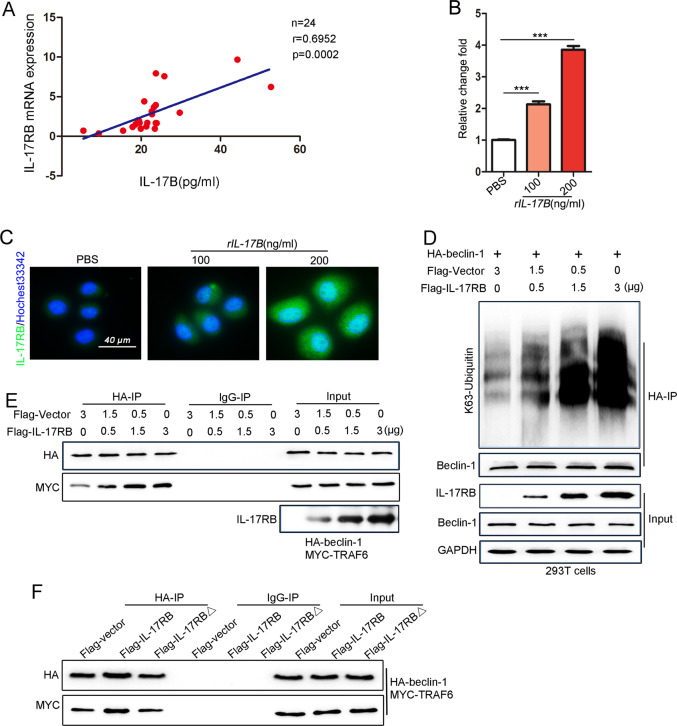
rIL-17B promoted autophagy activation and ubiquitination of Beclin-1 by inducing IL-17RB expression. **A** The relationship between IL-17B in the serum and the expression of IL-17RB mRNA in GC tissues from the same patient. **B** qRT-PCR was used to detect IL-17RB mRNA expression in MGC-803 cells treated with rIL-17B or PBS for 48 h (*n* = 3; ***p < 0.001). **C** Immunofluorescence was used to assay the IL-17RB protein expression in MGC-803 cells treated with rIL-17B or PBS for 48 h. **D** After transfection with different concentrations of Flag-IL-17RB and HA-Beclin-1 (2 μg) plasmids, 293T cells were subjected to IP using an HA antibody or control IgG, followed by IB with K63-linked ubiquitin, IL-17RB, Beclin-1, and GAPDH antibodies. **E** After transfection with different concentrations of Flag-IL-17RB, HA-Beclin-1, and MYC-TRAF6 plasmids, 293T cells were subjected to IP using an HA antibody or control IgG, followed by IB with HA, MYC, and IL-17RB antibodies. **F** Deletion of the TRAF6-binding domain in IL-17RB (Flag-IL-17RB△) abolished recruitment and binding between TRAF6 and Beclin-1.

Our previous study [8] revealed that GC tumor cells do not secrete IL-17B. Nevertheless, in the present study, the serum IL-17B level in GC patients was significantly higher than in healthy volunteers (Supplementary Fig. S12). Thus, we analyzed the relationship between the serum IL-17B level and IL-17RB mRNA expression in GC tissues; we found that the IL-17B level was positively correlated with IL-17RB expression in GC tissues (Fig. 8A). The results suggest that IL-17B is involved in regulating IL-17RB expression. We also discovered that exogenous IL-17B enhanced the expression of its receptor IL-17RB (Fig. 8B). The immunofluorescence results also indicate that IL-17B promoted IL-17RB expression on the membrane of MGC-803 cells (Fig. 8C).

Given the previous results, it is reasonable to assume that IL-17B induced IL-17RB expression, resulting in the K63-mediated ubiquitination of Beclin-1. Thus, we induced the overexpression of IL-17RB to different levels in 293T cells; the results revealed that with increasing IL-17RB expression, the level of the K63-mediated ubiquitination of Beclin-1 was significantly enhanced (Fig. 8D). IL-17RB overexpression also promoted direct binding between TRAF6 and Beclin-1 in a concentration-dependent manner (Fig. 8E). Huang et al. reported the importance of the TRAF6-binding domain of IL-17RB in mediating NF-κB signal transduction [6]. In the present study, deletion of the TRAF6-binding domain of IL-17RB abolished the binding between Beclin-1 and TRAF6 (Fig. 8F). The results suggest that IL-17RB induces K63-mediated Beclin-1 ubiquitination by recruiting TRAF6.

**Fig. 9:**
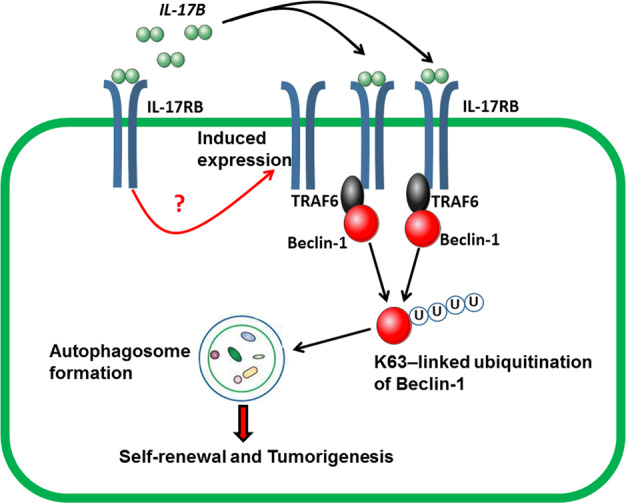
IL-17B induce the expression of IL-17RB, the underlying mechanism of which is not clear. The binding between IL-17B and IL-17RB recruits TRAF6 in cytoplasm to induce the K63-linked ubiquitination of Beclin-1, which leads the autopahagosome formation, to further reinforce the self-renewal and tumorigenesis ability of CSCs.

In summary, IL-17RB expression was high in spheroid cells and was closely correlated with the differentiation levels of GC tissues. IL-17B induced the expression of its receptor IL-17RB and activated IL-17RB downstream signaling cascades by promoting the recruitment of TRAF6 on IL-17RB. IL-17B/IL-17RB signaling initiated the direct binding of TRAF6 with Beclin-1, enhancing the K63-mediated ubiquitination of Beclin-1. Finally, IL-17B/IL-17RB signaling strengthened the self-renewal of CSCs by activating their autophagy and tumorigenesis (Fig. 9).

## Discussion

CSCs, a small subpopulation of tumor cells, are responsible for cancer initiation, propagation, metastasis, and recurrence. Accumulating evidence indicates that CSCs exist in various tumors [4]. Generally, CSCs are believed to account for most cancer-related death in GI cancer [29]. Besides, many studies have suggested that CSCs function by activating some key signaling pathways, including Wnt/β-catenin, Hedgehog-, Notch, and TGF-β-signaling [29, 30]. Some studies have proposed that the EMT is a critical regulator of CSC phenotypes [31]. Transcription factors—including Sox2, Oct4, Nanog, Klf4, and c-Myc—have been regarded to be involved in the maintenance of CSCs [32]. Even so, the molecular mechanisms behind the regulation of CSC homeostasis in cancer are not entirely understood. In the present study, we demonstrated that the IL-17B/IL-17RB signaling cascade plays a critical role in the homeostasis of CSCs.

The IL-17 family members are believed to be essential to the development of inflammatory diseases and some types of cancers. IL-17A, IL-17F, and IL-17C cause overlapping inflammatory responses, enhancing neutrophil-mediated immunity; IL-17E/IL-25 promotes Th2 immune responses and eosinophil activity [33]. However, knowledge of the biological functions of IL-17B remains limited. *IL-17B* gene expression was first identified in the pancreas, small intestine, and stomach in adults by using Northern blotting [34]. An early study suggested the pro-inflammatory function of IL-17B, which induces TNF-α release and exacerbates inflammatory arthritis [35]. Recent studies have provided evidence that IL-17B binds to its receptor to promote the antiapoptosis, tumorigenesis and the resistance to paclitaxel in breast cancer [6, 36] and to facilitate invasion, vasculogenic endothelial cell and macrophage recruitment to promote pancreatic cancer cell survival, enhancing tumor malignancy [7]. Studies have reported that IL-17B enhances invasion and metastasis in lung and thyroid cancer [37, 38]. Overall, the biological functions of IL-17B are yet to be clearly elucidated. IL-17B has been reported to play a role in chondrogenesis and osteogenesis in mouse embryonic limb buds [39], a process closely related to stem-cell differentiation and self-renewal. We propose the novel concept that IL-17B plays a critical role in the homeostasis of CSCs based on the following evidence obtained in the present study: (1) IL-17RB was highly expressed in LGR5^+^ CSCs, (2) IL-17B promoted the sphere-formation ability of CSCs in vitro and enhanced tumor growth, invasion and stemness in vivo, and (3) knockdown of IL-17RB in CSCs reversed the sphere-formation ability induced by IL-17B. Altogether, our findings indicate that IL-17B is an essential biological factor in the homeostasis of CSCs.

Our previous study suggested that IL-17B binds to its receptor IL-17RB, upregulating the expression of stem-cell markers in GC [8], and that exogenous IL-17B facilitates the adipogenic differentiation of human umbilical cord mesenchymal stem cells and GC-derived mesenchymal stem cells [40]. All the evidence regarding Beclin-1 ubiquitination indicates that IL-17B contributes to the regulation of the stemness of cancer cells, but the underlying molecular mechanisms are incompletely understood. In the present study, we demonstrated that IL-17B promoted IL-17RB expression, resulting in the enhanced physical interaction of TRAF6 with Beclin-1. Also, IL-17B promoted tumorigenesis and abdominal metastasis of CSCs in mouse models. Higher serum IL-17B levels were found in the clinical samples of patients with GC compared with those of healthy controls. All the evidence supports that IL-17/IL-17RB signaling activates autophagy by inducing Beclin-1 ubiquitination in CSCs.

Several lines of evidence indicate that IL-17A promotes the self-renewal of CD133^+^ cancer stem-like cells in ovarian cancer [41]. It also enables the transformation of quiescent gastric CSCs into invasive gastric CSCs [42], and may maintain the stemness of Nanog-positive CSCs in hepatocellular carcinoma [9]. Combining these findings with our findings, we propose that IL-17 family members possess a potentially crucial biological activity for mediating CSC functions. In addition, our results showed that IL-17B enhanced IL-17RB expression, suggesting that IL-17B and its receptor (IL-17RB) form a positive regulation loop for controlling CSC homeostasis. A previous study also reported that IL-17B treatment caused significant IL-17RB upregulation in lung cancer cells [37]. TGF-β1 released by Tregs in lymph nodes contributes to the upregulation of IL-17RB expression to promote breast cancer malignancy [43]. In the present study, we discovered that the expression of IL-17RB in gastric CSCs was significantly higher than that in non-CSCs, which is similar to the finding of one study that the expression of IL-17RB was higher in CD133^+^ ovarian cancer CSCs than in A2780 cells [41]. Thus, we conclude that high expression of IL-17RB in CSCs promotes cancer initiation, propagation, metastasis, and recurrence, and IL-17B and IL-17RB form a positive feedback loop that further amplifies the IL-17B/IL-17RB signaling cascade in the tumor environment. Therefore, targeting the IL-17B/IL-17RB signaling cascade is a novel approach for cancer treatment.

Autophagy has been extensively investigated in tumor biology and is generally believed to affect tumor metastasis, the EMT, drug resistance, and tumorigenesis [44–47]. However, how autophagy regulates CSC homeostasis remains unclear. Lee’s research group found that IL-17B signaling activates NF-κB by enhancing TRAF6 recruitment to IL-17RB [6]. Their study revealed that TLR4 recruits TRAF6 and promotes the K63-mediated ubiquitination of Beclin-1, which acts as a critical protein in autophagy activation [48]. Our previous results showed that IL-17B/IL-17RB signaling activates Akt [8], which activates the downstream classical signaling molecule mTOR [49], one of the most crucial signals for activating autophagy [50]. Therefore, we propose that IL-17B signaling activates autophagy to regulate the stemness of cancer cells. Because transcriptome sequencing analysis of HGC-27 cells treated with rIL-17B did not identify any molecules directly related to autophagy among genes with significant alterations (Supplementary Fig. S8C), we hypothesized that IL-17B might induce post-transcriptional modifications of key genes in autophagy. As expected, we demonstrated that IL-17B induced autophagosome formation in GC cells, and knockdown of ATG7 expression abolished the self-renewal of CSCs and expression of several CSC markers induced by IL-17B. In summary, on the basis of our results, we propose the novel mechanism that downstream activation of IL-17B/IL-17RB signaling activates autophagy, facilitating the self-renewal and tumorigenesis of GC cells, and this novel mechanism involves the recruitment of TRAF6 on the IL-17RB intracellular domain, which promotes direct binding of TRAF6 with Beclin-1 to enhance the K63-mediated ubiquitination of Beclin-1.

To identify the cell types with IL-17B expression, we performed single-cell RNA sequencing; however, the results did not reveal the cells types highly expressing IL-17B (data not shown). Guo et al. reported that IL-17RB knockdown in MOLM-13 AML cells had a stronger effect than IL-17B knockdown in vivo, reflecting the potential contribution of microenvironment-derived IL-17B [51]. We demonstrated a positive correlation between serum IL-17B level and IL-17RB expression in GC tissues in the present study. Recently, Yang et al. indicated that outer membrane vesicles derived from the identified commensal microbes induced IL-17B production to promote pulmonary fibrosis through TLR-MyD88 signaling [52]. Research has demonstrated that ~89% of new worldwide cases of GC can be attributed to *Helicobacterpylori* [53]. Therefore, it is possible that *H. pylori* could stimulate IL-17B secretion in the normal epithelial mucosa of the stomach away from the site of the primary GC tissue, leading to increased serum IL-17B levels in peripheral blood.

In summary, we demonstrated that IL-17B plays a novel function in controlling CSC homeostasis. We found that IL-17RB was highly expressed in GC-CSC-like cells, and that IL-17B promoted the sphere-formation ability of CSCs in vitro and enhanced tumor growth, invasion and stmness in vivo. Furthermore, autophagy activation was critically involved in the IL-17B/IL-17RB-mediated regulation of CSC functions. Altogether, the results revealed novel functions of IL-17B for CSCs. These functions indicate the importance of the IL-17B/IL-17RB signaling pathway for maintaining CSC homeostasis, suggesting that the IL-17B/IL-17RB signaling pathway is a new therapeutic target for cancer treatment.

## Materials and methods

### Human GC samples

Primary GC tissues and corresponding adjacent noncancerous tissues (i.e., tissues located more than 5 cm away from the primary site) were collected from patients with GC who underwent surgery at the Affiliated Hospital of Jining Medical University (Jining, China). We obtained 24 patient tissue specimens and the corresponding preoperative serum samples. All patient samples indicated GC. All GC tissue samples were obtained with the written consent of patients. This study was approved by the Clinical Ethics Committee of the Affiliated Hospital of Jining Medical University (No. 2018C004).

### Isolation of CD133^+^ HGC cells by magnetic bead sorting

Approximately 1 × 10^8^ HGC-27 cells were digested into single cells through trypsinization. CD133^+^ cells were isolated using the anti-human CD133-magnetic sorting antibody (Miltenyi Biotec, Gladbach, Germany). CD133^+^ cells would bind to the CD133-magnetic sorting antibody. The purity of CD133^+^ and CD133^−^ tumor cells was examined using BD FACSCanto II (BD Biosciences, San Diego, California, USA). Almost the same amounts of CD133^+^ and CD133^−^ tumor cells were stored in the Trizol reagent for further experiments.

### Recombinant human IL-17B treatment

Recombinant human IL-17B protein was purchased from R&D Systems (1248-IB-025), and was resuspended in PBS solution under sterile conditions. MGC-803 or HGC-27 cells were adherently cultured in the 6-wells plate, meanwhile add rIL-17B solution into the plate to the corresponding concentration, then incubator at 37 °C in an atmosphere of 5% CO_2_ for 48 h. To evaluate the effect of rIL-17B on the sphere-formation ability of GC cells, MGC-803 and HGC-27 cells treated with PBS or rIL-17B for 48 h, then the spheroid formation assay were operate.

### In vivo tumor xenograft model

MGC-803 cells were treated with the indicated concentrations of rIL-17B or PBS for 48 h and then 1 × 10^5^ cells per mouse were subcutaneously adoptively transferred into male BALB/c nude mice (*n* = 4 per group). These nude mice were aged 4–6 weeks and were obtained from the Laboratory Animal Center of Shanghai (Academy of Science, Shanghai, China). When the weight of the mice had decreased by ~20% and the diameter of the maximum tumor had reached about 3 cm, the mice were anesthetized and sacrificed. For tumor-initiating capacity analysis, different cell concentrations of HGC-27-shIL-17RB cells treated with 200 ng/ml rIL-17B and HGC-27-shControl cells treated with 200 ng/ml rIL-17B or PBS for 48 h (10^2^, 10^3^, 10^4^, and 10^5^ *n* = 8 for each cell concentration) were subcutaneously injected under the right lateral back of 6 weeks male NVSG nude mice, respectively. Eight days later, tumor formation was assessed. The tumor volume (*V*) was calculated using the following formula: *V* = 0.5 × *a* × *b*^2^ (where *a* denotes the longer tumor diameter and *b* the shorter tumor diameter). At the end of the experiment, the xenografts in each group were removed, weighed, and fixed in formalin for HE staining. Mice were randomly, blindly allocated to indicated groups according to a computer-generated randomization list. The Animal Ethics Committee approved all experimental protocols of the Affiliated Hospital of Jining Medical University (No. 2018B004).

### Statistical analysis

All data are presented as mean ± standard deviation. Statistically significant differences between groups were assessed using analysis of variance and Student’s *t* tests by employing Prism statistical analysis software (GraphPad, San Diego, USA); *p* < 0.05 was considered statistically significant.

A detailed description of the materials and methods used in this study is available in the online Supplementary Material. The information of antibody is listed in Supplementary Tables S1, S2, the primer sequences are listed in Supplementary Table S3, the shRNA and siRNA sequences are listed in Supplementary Table S4.

## Supplementary information

Supplementary materials -R3 clean version.

## Data Availability

The datasets used or analyzed during the current study are available from the corresponding author upon reasonable request.
