# Supplementary material for: IL-17B/IL-17RB signaling cascade contributes to self-renewal and tumorigenesis of cancer stem cells by regulating Beclin-1 ubiquitination
Source: Oncogene. 2021 Mar 1;40(12):2200–16. doi: 10.1038/s41388-021-01699-4 (PMC7994204; doi:10.1038/s41388-021-01699-4)
Supplement: Supplementary file 1 — Supplementary materials -R3 clean version. [file 41388_2021_1699_MOESM1_ESM.docx]

**Supplementary material：**

**IL17B/IL17RB signaling cascade contributes to self-renewal and tumorgenesis of gastric cancer stem cells by regulating Beclin-1 uniquitination**

**Materials and Methods**

**Cell culture**

All human-derived GC cell lines, namely HGC-27 and MGC-803, and 293T cells were obtained from the Institute of Biochemistry and Cell Biology at the Chinese Academy of Sciences (Shanghai, China). These cells were cultured in Dulbecco’s modified Eagle’s medium (DMEM) with high glucose concentration (Gibco, Grand Island, NY, USA). All media were supplemented with 10% fetal bovine serum (Gibco). The cells were cultured in a fully humidified incubator at 37°C in an atmosphere of 5% CO_2_ (Thermo, MA, USA). We tested for the presence of mycoplasma contamination before starting cellular experiments.

**Hematoxylin and eosin (HE) staining, immunohistochemistry, and immunofluorescence**

GC tissues were obtained from the operating room and were immediately stored in 4% paraformaldehyde for 48 h. These samples were processed into paraffin sections (5-μm thickness). Immunohistochemical staining was performed as described previously^28^. Primary antibodies and the related information are listed in Supplementary Table S1. Secondary antibodies were obtained from Boster (Wuhan, China). Images were sequentially acquired using a panoramic scanner (Panoramic 250/MIDI, 3DHISTECH, Hungary). Immunofluorescence was used to detect the expression of IL-17RB, HA (Beclin-1-tagged protein), and MYC (TRAF6-tagged protein). The fluorescent secondary antibodies and related information are listed in Supplementary Table S2. Images were sequentially acquired through light microscopy (Nikon, Tokyo, Japan) and confocal microscopy (GE, USA).

**Western immunoblotting**

Cell lysates were extracted in lysis buffer containing 50 mM sodium chloride, 1 mM ethylene glycol tetraacetic acid, 0.1% sodium dodecyl sulfate (SDS), 1 mM sodium fluoride, 1 mM sodium orthovanadate, 1 mg/mL aprotinin, and 1 mg/mL leupeptin in 10 mM Tris buffer (pH 7.4) and a standard proteinase inhibitor (1 mM n-phenylmethylsulfonyl fluoride). Equal amounts of total protein were separated on a 12% SDS–polyacrylamide gel and were transferred to nitrocellulose membranes (Millipore, Hong Kong, China). The membranes were incubated overnight with monoclonal antibodies against the selected primary antibodies (Supplementary Table S1). The membranes were washed three times with Tris-buffered saline and Tween and incubated with secondary antibodies (Supplementary Table S2) at room temperature for 1 h. The signals were visualized using a Luminata crescendo Western horseradish peroxidase substrate (Millipore, Billerica, MA, USA).

**Flow cytometry**

MGC-803 cells or HGC-27 cells were collected by trypsinization and were stained using antibodies, including anti-human IL-17RB-488, anti-human CD90-PE, anti-human CD133-PE, and anti-human LGR5-PE. These antibodies and related information are listed in Supplementary Table S1. Data were analyzed using FlowJo software. Forward and side-angle light scattering was used to ensure that only live cells were considered in the analysis. Gating was conducted using appropriate PE-conjugated or 488-conjugated isotypic control antibodies.

**RNA extraction, RT-PCR, and real-time RT-PCR (mRNA)**

Total RNA was isolated from cancer cells and tissues using the Trizol reagent (Invitrogen, Carlsbad, CA, USA). According to the manufacturer’s protocol; we reversed transcribed mRNA into cDNA (Vazyme Biotech Co., Ltd). Real-time quantitative RT-PCR (qRT-PCR) was conducted using the QuantiTect SYBR Green PCR kit (Toyobo, Japan). The primer sequences are listed in Supplementary Table S3.

**Lentiviral-mediated knockdown of target genes in cells**

The lentiviral expression vector (pLKO.1-puro) containing the shRNA sequence (Sigma, Shanghai, China) was selected for the target-specific gene silencing. The vector was infected into the cells according to the manufacturer’s instructions. The lentiviral vectors were produced using a lentivirus packaging mix (ViraPower, Invitrogen). Stablely infected cell lines were obtained after selecting with 1 μg/mL puromycin (Invitrogen) for 2 weeks. The efficiency of IL-17RB and ATG7 knockdown was evaluated using qRT-PCR and Western immunoblotting. The shRNA sequences of control and target-specific genes are listed in Supplementary Table S4.

**SiRNA-mediated knockdown of target genes in cells**

Chemically synthesized TRAF6 siRNAs and matching scrambled control siRNAs (NC) were obtained from GenePharma (Shanghai, China). According to the manufacturer’s instructions; the siRNAs were transfected into GC cells using the Lipofectamine 2000 reagent (Invitrogen) The siRNA sequences of control and target-specific genes are listed in Supplementary Table S4.

**Spheroid formation assay**

Differentially treated MGC-303 or HGC-27 cells were seeded into 6-well plates at indicated densities (Figure S1) and were grown in a serum-free medium consisting of H-DMEM basal media, 20 ng/mL EGF, 10 ng/mL bFGF, and 2% B27 (Gibco, New York, USA). In addition to culturing cells in serum-free media, spheroid cells were obtained using low adhesion (or ULA) tissue culture plates (Corning). The number of spheroids per well was counted under a light microscope at 40× magnification. The experiments were performed at least three times.

**Co-Immunoprecipitation**

The cells were lysed in a co-IP buffer. The cell lysate was added to 4 μL of HA antibody, placed on a 4°C shaker, and mixed overnight. In addition, the lysates were incubated with 25 μL of a protein A gel for 4 h at 4°C (Roche, Sweden). The precleared supernatant was then subjected to IP by centrifuging at 3000 rpm for 5 min. The collected protein complexes were washed six times with co-IP buffer and were analyzed using a Western blotting immunoassay.

**Peritoneal tumorigenesis and invasion experimental model**

After treatment with rIL-17B or PBS for 48h, 1×10^6^ MGC-803 cells per mouse were intraperitoneally injected into the mice, and the mice were subsequently inspected every 2 days and euthanized 1 month after injection. Solid tumors and organs were removed and examined. All experimental procedures involving animals were performed following the Guide for the Care and Use of Laboratory Animals (NIH publication no. 80–23, revised 1996).

**Enzyme-linked immunosorbent assay (ELISA) for serum IL-17B**

Serum samples were collected with all tissue specimens and were stored at −80°C until assayed. The serum IL-17B level was measured using a protein-specific ELISA kit (R&D Systems) following the manufacturer’s instructions. Each sample was measured in triplicate, and the mean concentration was calculated from a standard curve.

**Transcriptome Sequencing**

The RNA concentration and quality of HGC-27 cells that were treated with PBS or rIL-17B for 48h were assessed using a Nanodrop 2000 (Thermo Fisher Scientific, USA) and an Agilent 2100 Bioanalyzer (Agilent). Total RNA was sequenced using a HiSeqTM 2500 (Illumina) by Oebiotec (Shanghai, China). The data of transcriptome sequencing has been deposited in a public repository (Series record GSE164038). For information on GEO linking and citing, please refer to: <https://www.ncbi.nlm.nih.gov/geo/info/linking.html>.

**Supplementary Figures:**

**Figure S1.** Schematic diagram involved in the acquisition and re-differentiation of spheroid cells (related to Figure 1). (A) Workflow. HGC-27 (5×10^5^ cells) and MGC-803 cells (1×10^5^ cells) were cultured in 10 cm diameter dish containing 10 ml serum-free stem cell medium (formulation was listed in the Materials and Methods section) for 7 days. RNAs and proteins in the spheroid cells and adherent cells were extracted for follow-up studies. (B) and (C) qRT-PCR was performed for mRNA expression of *OCT4*, *NANOG*, *SOX2*, *LGR5*, *MUC1*, and *CK18* in spheroid cells and normal adherent cultured MGC-803 and HGC-27 cells (n = 3; ***p<0.001). (D) Workflow. Spheroid cells obtained in Panel A, were re-seeded in medium containing 10% FBS and cultured for 1 or 10 days, then RNA and protein were extracted at different time-points. (E) and (F) qRT-PCR was performed for analysis of *OCT4*, *NANOG*, *SOX2*, *LGR5*, *MUC1*, *CK18* mRNA expression in spheroid and re-adherent cultured MGC-803 and HGC-27 cells .

**Figure S2.** Representative flow cytometric analysis for IL-17RB, Lgr5, CD133 and CD90 in HGC-27 cells(A) and GC tumor tissue cells (B) (related to Figure 1).

**Figure S3.** (A) Representative HE staining for GC tissues with different degrees of differentiation. Representative immune-histochemical images for the level of IL-17RB, CK18 and Sox2 protein in GC tissues with different degrees of differentiation. HE and immuno-histochemical staining were performed for the serial sections of the same patient tissues. (B) qRT-PCR was performed for the analysis of *SOX2* mRNA expression in gastric cancer tissues with different degrees of differentiation (n = 22; *p<0.05; ***p<0.001).(C)The correlation of *IL-17RB*and *SOX2* mRNA expression(r= 0.6157, p=0023). (related to Figure 1).

**Figure S4.** (A and B) qRT-PCR (A) and Western blot (B) were performed to analyze the interference efficiency of lentivirus-based shIL-17RB knockdown in MGC-803 and HGC-27 cells. (C)The effects of IL-17RB knockdown on self-renewal capacity of MGC-803 cells (related to Figure 2).

**Figure S5.** The effect of rIL-17B on self-renewal in HGC-27 cells (related to Figure 2).(A)Representative images showing the spheroids of HGC-27 cells cultured in serum-free medium treated with different concentrations of rIL-17B or PBS for 7 days. (B) Statistical analysis for the number of spheroids (diameter > 50 μm) based on A (n=6, ***p<0.001). (C) Cell cycle analysis of HGC-27 cells treated with rIL-17B for 48 hours.

**Figure S6.** Representative flow cytometric analysis for Lgr5 (A), CD133 (B) and CD90 (C) expression on MGC-803 cells after treated with rIL-17B for 48h (related to Figure 2).

**Figure S7.** Statistical analysis for immunohistochemical staining of Cyclin-D1, Ki67, Sox9, Sox2 and CK18 in Figure 3F (n=4; **p<0.01; ***p<0.001) (related to Figure 3).

**Figure S8.** Transcriptome-sequencing analysis for the gene expression of HGC-27 cells treated with rIL-17B for 48h (related to Figure 5). (A) Heat map analysis for the expression of different genes in HGC-27 cells treated with 200ng/ml rIL-17B or PBS for 48h. (B) Ten key KEGG pathways. Different colors represent different p values, and the size of the dot represents the number of genes. (C) Top10 genes with the most obvious differences.

**Figure S9.** rIL-17B promoted self-renewal of GC cells by activating autophagy (related to Figure 5). (A and B) Western blot was performed for the expression of LC3, p62 and GAPDH in HGC-27(A) and 293T cells (B) treated with rIL-17B or not. (C) The expression of ATG7, LC3, Sox2, Oct4 and GAPDH was analyzed by Western blot in shATG7 or shControl infected MGC-803 cells. (D) The expression of ATG7, Sox2, Oct4 and GAPDH was analyzed by Western blot in shATG7 infected MGC-803 cells treated with rIL-17B.

**Figure S10.** Chloroquine (CQ) blocked the transformation of LC3 and self-renewal induced by rIL-17B (related to Figure 5). (A) Western blot was performed for the expression of LC3, p62 and GAPDH in rIL-17B or/and 25nmCQ treated MGC-803 cells for 48h.(B) Representative images showing the spheroids of MGC-803 cells treated with rIL-17B or/and 25nm CQ for 48h, then cells were cultured in serum-free medium for seven days. (C) Statistical analysis the number of spheroids (diameter > 50 μm) according to Figure B (n = 6; **p<0.01).

**Figure S11.**The expression of Beclin-1 in rIL-17B-treated MGC-803 cells was analyzed by Western blot (A) and qRT-PCR (B) (related to Figure 6)**.**

**Figure S12.**ELISA analysis for the levels of IL-17B protein in the serum of gastric cancer patients and normal volunteers (n = 24; ***p <0.001).

Supplementary Table S1: Primary Antibodies

| Primary Antibody | Source | Detection and dilution ratio | Manufacturer |
| --- | --- | --- | --- |
| GAPDH | Rabbit | WB1:10000 | Bioworld |
| IL-17RB | Rabbit | WB1:800/IHC1:100/IF1:100/Flow1:200 | Abcam |
| SOX9 | Rabbit | IH:1:1000 | Merck Millipore |
| LC3 | Rabbit | WB1:1000 | Cell signaling technology |
| Oct4 | Mice | WB1:1000 | Cell signaling technology |
| TRAF6 | Rabbit | WB1:1000 | Cell signaling technology |
| ATG7 | Rabbit | WB1:1000 | Cell signaling technology |
| Beclin-1 | Rabbit | WB1:1000 | Cell signaling technology |
| Ki67 | Rabbit | WB1:1000 | Cell signaling technology |
| Cyclin-D1 | Rabbit | WB1:1000 | Cell signaling technology |
| P62 | Rabbit | WB1:1000 | Proteintech |
| Sox2 | Mice | WB1:1000/IHC1:100 | Cell signaling technology |
| CK14 | Rabbit | WB1:500 | Bioworld |
| CK18 | Rabbit | IHC1:50 | Bioworld |
| HA-tag | Rabbit | IP:1:200 | Cell signaling technology |
| HA-tag | Mice | WB1:1000 | Cell signaling technology |
| MYC-tag | Goat | WB1:1000 | Cell signaling technology |
| Ubiquitin | Mice | WB1:1000 | Cell signaling technology |
| K63-Ubiquitin | Mice | WB1:1000 | LifeSpan BioSciences |
| PE anti-human CD90 Thy1 Antibody | Rabbit | Flow 1:200 | Biolegend |
| PE anti-human CD133 Antibody | Rabbit | Flow 1:200 | Biolegend |
| PE anti-human LGR5 GPR49 Antibody | Rabbit | Flow 1:200 | Biolegend |
| 488 anti-human IL-17RB Antibody | Rabbit | Flow 1:200 | Biolegend |
| PE Mouse IgG1, kappa Isotype | Rabbit | Flow 1:200 | Biolegend |

Supplementary Table S2: Second Antibodies

| Second Antibody | Detection and dilution ratio | Manufacturer |
| --- | --- | --- |
| Anti-Rabbit antibody-HRP | WB1:2000 | Cell signaling technology |
| Anti-Mice antibody-HRP | WB1:2000 | Cell signaling technology |
| Anti-Goat antibody-HRP | WB1:2000 | Cell signaling technology |
| Anti-Rabbit antibody-antibody-Alexa Fluor 647 | IF 1:1000 | Cell signaling technology |
| Anti-Mice antibody-Alexa Fluor 488 | IF 1:1000 | Cell signaling technology |
| Anti-Goat antibody-Alexa Fluor 555 | IF 1:1000 | Abcam |

Supplementary Table S3. Sequences of real-time PCR primers

| mRNA | Primer | Sequences (5′-3 ′) | Annealing temperature |
| --- | --- | --- | --- |
| Human-IL-17RB | Forward | CTTGGTGGCCTTCAACAAGC | 60°C |
|  | Reverse | AGAGCCGACCGTTCAATGTG |  |
| Human-MUC1 | Forward | TCTCAGACGTCAGCGTGAGT | 60°C |
|  | Reverse | CTCGCTCATAGGATGGTAGG |  |
| Human-CK14 | Forward | GATGACTTCCGCACCAAGTA | 60°C |
|  | Reverse | TCTCCTCCTCGTGGTTCTTC |  |
| Human-CK18 | Forward | CAGGACCTCGCCAAGATCAT | 63°C |
|  | Reverse | GTTCTCCAAGCTGGCCTTCA |  |
| Human-Sox2 | Forward | ACACCAATCCCATCCACACT | 60°C |
|  | Reverse | GCAAACTTCCTGCAAAGCTC |  |
| Human-Oct4 | Forward | TTGAGGCTCTGCAGCTTAG | 60°C |
|  | Reverse | GCCGGTTACAGAACCACAC |  |
| Human-Nanog | Forward | CCTGATTCTTCCACCAGTCC | 60°C |
|  | Reverse | TGCTATTCTTCGGCCAGTTG |  |
| Human-β-actin | Forward | GACCTGTACGCCAACACAGT | 59°C |
|  | Reverse | CTCAGGAGGAGCAATGATCT |  |

Supplementary Table S4. ShRNA or siRNA Oligonucleotides

| Target Gene | Sequences (5′-3 ′) |
| --- | --- |
| Human-IL-17RB | Forward CCGGGATGCTACAACATGATCTAATCTCGAGATTAGATCATGTTGTAGCATCTTTTTG |
|  | Reverse AATTCAAAAAGATGCTACAACATGATCTAATCTCGAGATTAGATCATGTTGTAGCATC |
| Human-ATG7 | Forward CcgggcCTGCTGAGGAGCTCTCCATCTCGAGATGGAGAGCTCCTCAGCAGGCTTTTTg |
|  | Reverse aattcaaaaagcCTGCTGAGGAGCTCTCCATCTCGAGATGGAGAGCTCCTCAGCAGGC |
| Human-TRAF6 (siRNA) | Forward GCAGUGCAAUGGAAUUUAUTT |
|  | Reverse AUAAAUUCCAUUGCACUGCTT |
| Negative control(siRNA) | Forward UUCUCCGAACGUGUCACGUTT |
|  | Reverse ACGUGACACGUUCGGAGAATT |
| GAPDH Positive control(siRNA) | Forward UGACCUCAACUACAUGGUUTT |
|  | Reverse AACCAUGUAGUUGAGGUCATT |
